# Serial measurements of Paraoxonase-1 (PON-1) activity in horses with experimentally induced endotoxemia

**DOI:** 10.1186/s12917-020-02629-4

**Published:** 2020-11-04

**Authors:** Donatella Scavone, Micaela Sgorbini, Alexandre S. Borges, José P. Oliveira-Filho, Valentina Vitale, Saverio Paltrinieri

**Affiliations:** 1 Department of Veterinary Medicine, Veterinary Teaching Hospital, University of Milan, Lodi, Italy; 2 Department of Veterinary Sciences, Veterinary Teaching Hospital, Univeristy of Pisa, Pisa, Italy; 3 Department of Veterinary Clinical Science, School of Veterynary Medicine and Animal Sicence, Sao PAulo State University (Unesp), Botucatu, Brazil; 4 School of Veterinary Science, Veterinary Teaching Hospital, University of Sydney, Camden, NSW Australia

**Keywords:** Equine, Inflammation, Oxidative stress

## Abstract

**Background:**

Paraoxonase-1 (PON-1) is an antioxidant enzyme, whose activity decreases during the acute phase response in many species. Little is known about PON-1 and its role as a negative acute phase protein during septic inflammation in horses, but promising findings about its utility in diagnosing SIRS and predicting the outcome in diseased horses, were recently highlighted. The objective of the study was to investigate the behaviour of PON-1 in horses after experimentally induced endotoxemia. To this aim, PON-1 activity was measured on 66 plasma samples collected from six clinically healthy mares, previously included in another study, before and at multiple time points between 12 and 240 h after intravenous infusion of *Escherichia coli* O55:B5 lipopolysaccharide (LPS).

**Results:**

Compared with baseline values, a progressive transient decrease of PON-1 activity was observed starting from 24 h post-infusion, with lowest values observed between 3 to 7 days post-infusion, followed by a normalisation to pre-infusion levels the tenth day.

**Conclusions:**

The results of this study suggest that measurement and monitoring of PON-1 activity might be useful to evaluate progression and recovery from endotoxemia in horses. Further studies in horses with naturally occurring sepsis are warranted.

**Supplementary Information:**

The online version contains supplementary material available at 10.1186/s12917-020-02629-4.

## Background

Paraoxonase-1 (PON-1) was the first discovered and the most studied of the three members of the mammalian paraoxonases family [1, 2]. This enzyme, with esterase and lactonase activities, is mainly synthesized in the liver, circulates in blood bound to high-density lipoproteins (HDL) [3] and protects low-density and high-density lipoproteins from oxidation by hydrolysing oxidized lipids [4–8]. PON-1 is therefore considered an antioxidant enzyme, but also a modulator of the inflammatory response [9] and part of the immune system [10]. In the last year’s research, there has been growing interest about the role of PON-1 in the pathogenesis of many diseases characterized by oxidative stress, primarily in human medicine [11, 12], but more recently also in veterinary medicine. In particular, several studies have highlighted the potential of PON-1 in the pathogenesis, diagnosis and prediction of outcome in naturally occurring and experimentally induced sepsis in many species, including horses [13–25]. During the acute phase response, changes in lipoprotein occur [26] and PON-1 in HDL is replaced by acute phase proteins, mainly serum amyloid A. Together with down-regulation of hepatic PON-1 gene expression, these changes cause a decrease in its activity [27], which appears to be more pronounced when inflammation is associated to infection [10] and when inflammation is more severe [16]. A recent study validated a paraoxon-based method to measure serum PON-1 activity in horses and provided reference intervals in healthy animals for this species [28]. Interestingly, in a further study, low PON-1 activity was reported only in some of the horses with systemic inflammatory response syndrome (SIRS) [15]. Therefore, although the latter study indicated that low PON-1 activity at admission may predict the presence of SIRS or a poor outcome, to date very little is known about PON-1 in horses, and additional information is required to confirm that this protein could have a diagnostic or prognostic marker, as reported for other species. The aim of the study was to investigate the behaviour of plasma PON-1 activity from horses with experimentally induced endotoxemia included in a previous study, on which the administration of LPS induced evident haematological and biochemical changes consistent with inflammation. The hypothesis of the study was that oxidative phenomena associated with endotoxemia may induce a decrease of PON-1 activity that parallels the other changes detected after LPS administration.

## Results

**Fig. 1:**
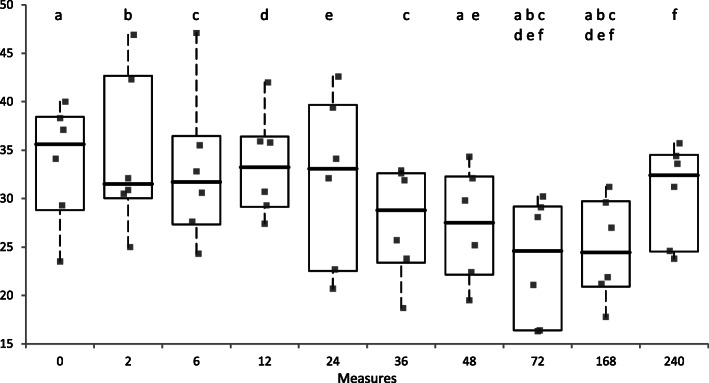
Plasma PON-1 activity (U/mL) measured at scheduled time points before (T0) and after (T2-T240) LPS administration in individual healthy horses (*n* = 6). The boxes indicate the I-III interquartile range (IQR), the horizontal black line indicate the median values, whiskers extend to further observation within quartile I minus 1.5 × IQR or to further observation within quartile III plus 1.5 × IQR. Time points that are significantly different to each other are indicated by the same letters on the top of boxplots

Table 1 and Fig. 1 report PON-1 activity measured at scheduled time points. A progressive decrease in PON-1 activity from baseline was observed from 24 h after the end of LPS infusion.

**Table 1:** Mean, standard deviation, median and range of plasma PON-1 activity for scheduled time points before (T0) and after (T2-T240) LPS administration in healthy horses (*n* = 6). Asterisks indicate values significantly different (*P* < 0.05) from reported time points

Blood sampling time (hours)	PON-1 activity (U/mL)		
	Mean ± S.D.	Median	Min-Max range
0	33.7 ± 6.3	35.6	28.8–38.4
2	34.6 ± 8.2	31.5	30.0–42.7
6	33.0 ± 7.9	31.7	27.3–36.5
12	33.5 ± 5.4	33.3	29.1–36.4
24	31.9 ± 8.8	33.1	22.5–39.7
36	27.6 ± 5.8	28,8	23.4–32.6
48	27.2 ± 5.8	27.5	22,2-32,3
72	23.5 ± 6.4	24.6	16.4–29.2
168	24.8 ± 5.3	24,5	20.9–29.7
240	30.6 ± 5.1	32.4	24.5–34.5

The Friedmann test evidenced that results collected at the different sampling times were significantly different to each other (*P* < 0.001). Mean PON-1 values measured from 48 h to 168 h post-infusion were significantly lower than those recorded in the samples collected in the first 24 h and especially from the baseline values. After this transient decrease, at 240 h post-infusion PON-1 activity returned to similar baseline values, significantly higher than lowest ones observed 72- and 168-h post-infusion. Moreover, PON-1 activity was negatively correlated with fibrinogen (*P* < 0.001, rs = − 0.899) and positively correlated with the PCV (*P* = 0.008; rs = 0.779). No other significant correlations with laboratory parameters were found. Similarly, the clinical score was not correlated with PON-1 activity.

## Discussion

PON-1 has an established role in modulating oxidative stress, which is a major promoter and mediator of the systemic inflammatory response occurring in sepsis [17]. The activity of PON-1 during septic inflammation has been evaluated in other species [18, 20, 25, 29, 30], but not in horses yet. We investigated plasma PON-1 activity following intravenous injection of 30 ng/kg of LPS (*E. coli* O55:B5) in healthy horses included in a previous study [31]. The authors of the previous study demonstrated significant clinical changes caused by endotoxemia within the first 12 h post-infusion, significant changes in WBC counts 6 h and marked neutrophilia at 24 h after LPS infusion. Then, all these changes normalized in all horses after 48 h. Plasma iron concentration significantly decreased to lowest values at 12 h post-infusion and raised again starting from 24 h, returning to baseline values at 36 h. As expected, we observed a decrease in PON-1 activity. Differently from clinical signs and changes in WBC and plasma iron reported in the previous study [31], PON-1 activity recorded in the present study did not show significant changes in the first 12 h. On the contrary, a significant decrease in PON-1 activity was recorded starting from 48 h post-infusion, when all the other clinical and analytical parameters had returned to normal reference intervals. The simultaneous decrease in PON-1 activity and recovery from clinical signs is in contrast with those studies that in humans reported PON-1 increase when patients with naturally occurred sepsis clinically improved [18, 19, 22]. The same studies however [19, 22], reported a sustained decrease in PON-1 activity for several days, which we also observed. Our study is based on an experimental model of endotoxemia, which does not completely mimic the conditions and pathologic pathways of naturally occurring endotoxemia. In the previous study [31] the evaluation of short-term effects of LPS infusion (i.e. 0–6 h after LPS administration) was based on clinical changes and not on a complete SIRS score, that also includes the evaluation of WBC count. However, all the horses had at least 2 alterations of clinical parameters consistent with SIRS status evaluation proposed by Roy et al. [32] and therefore it can be assumed that LPS administration induced a systemic inflammatory reaction. Our results agree with other papers assessing PON-1 activity in experimentally induced endotoxemic animals. In dogs, Tvarijonaviciute et al. [20] reported normalisation of clinical signs in 48 h after LPS administration (although clinical signs occurred earlier compared with horses included in our study), and decreased PON-1 activity at 30 h, which progressed and became significant at 48 h compared to 24 h; a similar trend to what we observed. These authors did not measure PON-1 activity over 48 h post LPS administration, so a comparison of long-term normalisation of PON-1 activity cannot be made. Feingold et al. [27] found in Syrian hamsters a progressive decrease in PON-1 activity, already significant 8 h after LPS administration, which even in this case persisted at least up to the last sampling at 48 h. These and our results seem to state that experimentally induced endotoxemia induces changes in PON-1 activity that does not have a rapid resolution, contrarily to clinical signs, that normalize earlier. This latter discrepancy may explain the lack of significant correlations between PON-1 activity and the clinical score. Our data seem to state that endotoxemia triggers changes in PON-1 activity later and for a longer period, compared with leukocyte count and plasma iron concentration. Again, this delayed response may explain the lack of correlations between PON-1 activity and many laboratory changes, except for fibrinogen that, being a positive acute phase protein, negatively correlated, as expected, with PON-1 activity and for the PCV that increased soon after LPS infusion likely due to dehydration and then decreased to normal values, thus paralleling the late decrease of PON-1. This late PON-1 response could be interpreted as a delayed occurrence of inflammatory oxidations and oxidative stress, possibly due to a different PON-1 metabolism during inflammation in horses compared to other species, as suggested by Ruggerone et al. [15]. Otherwise, it may also be possible that anti-inflammatory oxidations occurred in the first 12 h post-LPS infusion, determining PON-1 activity fluctuations not wide enough to produce significant changes, possibly because of a less severe oxidative stress in horses compared to other species (Ruggerone et al., 2020). A reduced hepatic synthesis, due to cytokine down regulation or to liver function impairment after LPS administration, could have determined the subsequent noticeable decrease of PON-1 activity from the third day up to the seventh. Feingold et al. [27] simultaneously evaluated also liver PON-1 mRNA expression and observed an earlier and greater decrease in mRNA level, starting from 4 h post-LPS administration and reaching lowest values at 16 h, followed by a progressive increase which did not reach baseline values at 48 h. This led to the hypothesis that acute changes in PON-1 production mildly affect serum levels, possibly because of a relatively long half-life of the enzyme [27]. Liver expression of PON-1 mRNA was not evaluated in our study, but a similar mechanism would explain the delayed and sustained decrease in PON-1 activity that followed clinical recovery and normalisation of other laboratory parameters. Investigation of PON1 liver expression in the future may clarify the causes of delayed and prolonged decrease of PON-1. Furthermore, PON-1 activity is dependent on the lipid and protein composition of HDL [33], and correlation with lipid profile and other markers of inflammation such as serum amyloid A, may help acquiring knowledge about PON-1 metabolism in this species.

Biomarkers of sepsis so far evaluated in horses include interleukin-1β (IL-1β), interleukin-10 (IL-10), interleukin-6 (IL-6), serum amyloid A (SAA), soluble CD14 (sCD14) and procalcitonin [34, 35]. Measurement of sCD14 provided contrasting results: no significant differences in concentration were recorded among time in experimental settings [34], while sCD14 was higher in in spontaneous clinically endotoxemic than nonendotoxemic horses, showing however no correlation with LPS concentration [36]. In many studies involving different types of experimentally induced endotoxemia in horses, cytokine expression was rapidly upregulated. Following LPS infusion, IL-1β and IL-6 gene expression peaked at 60 and 90 min respectively and decreased to still significantly higher levels than baseline 3 h post infusion [37]. In a similar way, serum TNF-α increases within 30 min, peaks at 60 min, and decreases 2–3 h from endotoxin administration [37, 38]. Even if studies about SAA in experimentally induced endotoxemic horses are not available, blood SAA concentration is reported to increase up to 1000 times 6 h after inflammatory stimuli and decrease within 12 h after stimulus removal [39–41]. It is not known if infection causes an earlier or persistent increase in SAA concentration compared with non-infectious inflammation, but there is evidence that infections are particularly effective in producing strong SAA responses [42], especially in neonatal sepsis [43]. Since SAA is produced by the liver in response to cytokines stimulation, it is reasonable to think its increase in circulation be delayed. A decrease in PON-1 activity would be expected together with the early increase in SAA levels, since during the acute phase response the latter displaces PON-1 in HDL [44] and these two acute phase proteins were reported to be inversely correlated [45]. However, cytokine-dependent hepatic regulation could have major impact on PON-1 activity rather than displacement by SAA [46], and this would explain the delayed decrease of PON-1 activity, as reported above. Comparing changes in PON-1 activity detected in this study and changes in other markers investigated in experimentally-induced endotoxemic horses, it seems that other markers normalise before PON-1 decreases. In fact, CCL-2 and IL-10 concentrations increased, peaked and decreased within 24 h post-infusion. Procalcitonin as well showed a similar trend, but its concentration, even if decreased, was still statistically higher than baseline 24 h post-infusion [34]. However, endotoxin is a single factor implicated in the development of sepsis, and it is well known that differences exist between experimentally-induced and clinical endotoxemia and sepsis [47]. In humans, PON-1 activity and CCL-2 concentration have an inverse correlation during natural infection [48] and CCL-2 decreases together with the increase of PON-1 and the resolution of naturally occurring septic processes after several days of hospitalisation [19]. Investigation and comparison of changes in these markers during natural infection in horses would clarify their behaviour, relationship and usefulness in this species. This study has some limitations. Firstly, the long storage of samples that may have in part affected the magnitude of PON-1 activity. A previous study demonstrated that 6 months storage at − 20 °C can artifactually slightly increase PON-1 activity in dogs [49]. However, no information on storage stability at − 80 °C, or storage studies on equine PON-1 are available. However, as a support of the hypothesis of a storage effect, PON-1 activity was lower than the reference intervals [28] in most of the horses at T0 despite the lack of any clinical or laboratory abnormality associated with inflammation. Nevertheless, this effect, if any, was present for all the samples included in this study and despite possible storage artifacts, fluctuations of PON-1 activity consistent with endotoxin-induced inflammation were present. A second limitation is the low caseload, coupled with the lack of a control group inoculated with a sham solution: this approach, that however, is in line with other studies based on a complex study design and is supported also by ethical reasons. However, also in this case, such a low but well standardized caseload, on which post-inoculation results were compared with baseline values recorded before LPS administration, allowed to have a better overview of the possible effect of LPS infusion on PON-1 activity compared with in field studies, on which several variables cannot allow to evaluate the direct effect of sepsis on clinical and laboratory parameters. Undoubtedly, following an inflammatory stimulus of septic type, such as the administration of endotoxins, PON-1 activity decreases, as reported for other species. According to our results, plasma iron concentration appears to be an earlier marker of sepsis onset and positive resolution compared to PON-1 activity. However, all horses showed a normalisation of PON-1 activity to baseline values 10 days after LPS administration. As claimed by Oliveira-Filho et al. [31], the inflammatory model adopted by the authors is safe and efficient and caused an acute systemic inflammation without prolonged inflammatory effects. As noted above, in naturally occurring sepsis, clinical and pathological conditions are not as well standardised. Therefore, in natural situations, where the extent and severity of inflammation [17] are very variable, PON-1 might prove to be useful as a diagnostic or prognostic marker of sepsis. In this case, measurement of PON-1 activity could be useful where limitations of iron measurement in detecting systemic inflammation exist, for example during corticosteroid administration, iron supplementation, haemolysis and age-relate variability [50].

## Conclusion

This is the first study reporting PON-1 activity in horses with ongoing endotoxemia caused by experimental LPS administration. As expected, LPS infusion induced a progressive transient decrease of PON-1 activity, which became maximum from 3 days to 7 days post-infusion, followed by a normalisation to pre-infusion levels the tenth day. The inflammation model employed in the study does not mimic the variety and severity of septic diseases occurring in the clinical practice. Therefore, further studies are warranted to investigate changes in PON-1 activity during natural onset of endotoxemia in horses. However, our data suggest that measurement and monitoring of PON-1 activity might be useful to evaluate progression of the septic process and recovery in endotoxemic horses and possibly to diagnose it.

## Methods

The study was performed on 66 plasma samples from six non-pregnant 6–9 years old clinically healthy Quarter Horse mares, with mean body weight 425 (± 20) kg, previously sampled during another study [31]. The horses belonged to the São Paulo State University (Unesp), School of Veterinary Medicine and Animal Science, herd. The study was submitted and approved by the São Paulo State University, Institutional Animal Care and Use Committee Institutional Animal Care and Use Committee (108-A/2007). All experiments on animals were carried at the São Paulo State University (Unesp).

In these animals, endotoxemia was induced by intravenous infusion of 30 ng/kg of LPS (*E. coli* O55:B5, Sigma-Aldrich, St. Louis, MO, USA) in 300 ml of 0.9% sterile NaCl over 30 min, with blood samples collected at time 0 h (immediately before LPS infusion) and at several time intervals from 2 to 240 h after infusion. Blood samples were collected through a 14G catheter aseptically inserted in the right jugular vein. Heparinized samples were used to perform a cell blood count (only at some time point) and then centrifuged. Aliquots of plasma were used to measure total protein, serum iron and fibrinogen, and then stored at − 80 °C until the analysis. Part of the aliquots were transferred on dry ice to the University of Milan for the measurement of PON-1. Details of the haematological and biochemical parameters that were investigated in the previous study [31], as well as the clinical score and laboratory results recorded at the different time points selected for the current study are reported as supplementary material (additional file table). To summarize, all horses subjected to LPS infusion showed clinical signs related to endotoxemia, such as depression, muscle fasciculations, intestinal hypomotility and mild-to-moderate abdominal pain and they had a significant increase in the clinical score proposed by Moore et al. [51] based on heart and respiratory rate and of body temperature from 1 to 6 h (*P* < 0.01). Starting from 12 h and up to 240 h post-infusion, clinical score was reported as normal. Conversely, neutrophilic leucocytosis, lymphopenia and decreased plasma iron were detectable between 6 and 24 h, then gradually normalized over time, with the return to normal levels at 72 h [31]. All animals showed no sequelae at the end of the study and returned to the activities they performed before the study.

On the basis of clinical score and laboratory data, the current study was done on samples collected at time 0, 2, 6, 12, 24, 36, 48, 72, 168, and 240 h after infusion, to concentrate the analysis in samples that had the most prominent clinical or laboratory changes and to include two additional samples (168 and 240 h) to assess possible long term effects on PON-1 activity.

Plasma PON-1 activity was measured spectrophotometrically using an automated analyser (Cobas Mira, Roche diagnostic, Basel, Switzerland), and the enzymatic method already validated in horses [28]. Briefly, 6 μL of samples were incubated at 37 °C with 89 μL of distilled water and 100 μl of reaction buffer (glycine buffer 0.05 mM, pH 10.5 containing 1 mM of paraoxon-ethyl, purity > 90% [Sigma-Aldrich, Saint Louis, MO, USA], and 1 mM of CaCl_2_). The rate of hydrolysis of paraoxon to p-nitrophenol was measured by monitoring the increase in absorbance at 504 nm using a molar extinction coefficient of 18.050 L· mol^− 1^ · cm^− 1^ as previously suggested [27]. The unit of PON-1 activity expressed as U/mL is defined as 1 nmol of p-nitrophenol formed per minute under the assay conditions.

Statistics were run with Analyse-it v 5.01 (Analyse-it Software Ltd., Leeds, United Kingdom) statistical analysis add-in software for Excel (Redmond, WA, USA). Since the Shapiro-Wilk test demonstrated that data were not normally distributed, data recorded in the different sampling times were compared to each other using a non-parametric ANOVA test for repeated measurements (Friedmann test) followed by the Wilcoxon signed rank test to compare paired results recorded at each sampling time. The Spearman test was used to investigate the possible correlation between mean PON-1 activity and mean values of the clinical or laboratory changes recorded in the previous study [31]. Statistical differences were set for *P* < 0.05.

## Supplementary Information

**Additional file 1.** Mean ± standard deviation regarding the clinical score and the main clinico-pathological changes recorded in the previous study [31] in the time points selected for inclusion in this study.

## Data Availability

Raw data are available upon request. The datasets used and/or analyzed during the current study are available from the corresponding author on reasonable request.

## Abbreviations

HDL: High-density lipoproteins
LPS: Lipopolysaccharide
PON-1: Paraoxonase-1
SIRS: Systemic inflammatory response syndrome

## References

[1] Primo-Parmo SL, Sorenson RC, Teiber J, La Du BN (1996). The human serum paraoxonase/arylesterase gene (PON1) is one member of a multigene family. Genomics..

[2] Costa LG, Furlong CE (2002). Paraoxonase (PON1) in health and disease: basic and clinical aspects.

[3] Camps J, Marsillach J, Joven J (2009). The paraoxonases: role in human diseases and methodological difficulties in measurement. Crit Rev Clin Lab Sci.

[4] Mackness MI, Arrol S, Durrington PN (1991). Paraoxonase prevents accumulation of lipoperoxides in low-density lipoprotein. FEBS Lett.

[5] Watson AD, Berliner JA, Hama SY, La Du BN, Faull KF, Fogelman AM (1995). Protective effect of high density lipoprotein associated paraoxonase. Inhibition of the biological activity of minimally oxidized low density lipoprotein. J Clin Invest.

[6] Aviram M, Rosenblat M, Bisgaier CL, Newton RS, Primo-Parmo SL, La Du BN (1998). Paraoxonase inhibits high-density lipoprotein oxidation and preserves its functions. A possible peroxidative role for paraoxonase. J Clin Invest.

[7] Shih DM, Gu L, Xia YR, Navab M, Li WF, Hama S (1998). Mice lacking serum paraoxonase are susceptible to organophosphate toxicity and atherosclerosis. Nature..

[8] Rozenberg O, Rosenblat M, Coleman R, Shih DM, Aviram M (2003). Paraoxonase (PON1) deficiency is associated with increased macrophage oxidative stress: studies in PON1-knockout mice. Free Radic Biol Med.

[9] James RW (2006). A long and winding road: defining the biological role and clinical importance of paraoxonases. Clin Chem Lab Med.

[10] Camps J, Iftimie S, García-Heredia A, Castro A, Joven J (2017). Paraoxonases and infectious diseases. Clin Biochem.

[11] Marsillach J, Parra S, Ferré N, Coll B, Alonso-Villaverde C, Joven J, Mackness B, Mackness M, Aviram M, Paragh G (2008). Paraoxonase-1 in chronic liver diseases, neurological diseases and HIV infection. The paraoxonases: their role in disease development and xenobiotic metabolism.

[12] Mackness M, Mackness B, Komoda T (2014). Current aspects of paraoxonase-1 research. The HDL handbook- biological functions and clinical implications.

[13] Turk R, Habuš J, Flegar-Meštrić Z, Svetina A, Mojčec V, Perkov S (2011). Serum platelet-activating factor acetylhydrolase and paraoxonase-1 activity in horses infected with Leptospira spp. Acta Trop.

[14] Radakovic M, Davitkov D, Borozan S, Stojanovic S, Stevanovic J, Krstic V (2016). Oxidative stress and DNA damage in horses naturally infected with Theileria equi. Vet J.

[15] Ruggerone B, Paltrinieri S, Giordano A, Scavone D, Nocera I, Rinnovati R (2020). Paraoxonase-1 activity evaluation as a diagnostic and prognostic marker in horses and foals. J Vet Intern Med.

[16] Chuang C-C, Shiesh S-C, Chi C-H, Tu Y-F, Hor L-I, Shieh C-C (2006). Serum total antioxidant capacity reflects severity of illness in patients with severe sepsis. Crit Care.

[17] Draganov D, Teiber J, Watson C, Bisgaier C, Nemzek J, Remick D (2010). PON1 and oxidative stress in human sepsis and an animal model of sepsis. Adv Exp Med Biol.

[18] Novak F, Vavrova L, Kodydkova J, Novak FS, Hynkova M, Zak A (2010). Decreased paraoxonase activity in critically ill patients with sepsis. Clin Exp Med.

[19] Sans T, Rull A, Luna J, Mackness B, Mackness M, Joven J (2012). Monocyte chemoattractant protein-1 and paraoxonase-1 and 3 levels in patients with sepsis treated in an intensive care unit: a preliminary report. Clin Chem Lab Med.

[20] Tvarijonaviciute A, Kocaturk M, Cansev M, Tecles F, Ceron JJ, Yilmaz Z (2012). Serum butyrylcholinesterase and paraoxonase 1 in a canine model of endotoxemia: effects of choline administration. Res Vet Sci.

[21] Li Y, Zhai R, Li H, Mei X, Qiu G (2013). Prognostic value of serum paraoxonase and arylesterase activity in patients with sepsis. J Int Med Res.

[22] Bojic S, Kotur-Stevuljevic J, Kalezic N, Jelic-Ivanovic Z, Stefanovic A, Palibrk I (2014). Low paraoxonase 1 activity predicts mortality in surgical patients with sepsis. Dis Markers.

[23] İnal V, Yamanel L, Taşkın G, Tapan S, Cömert B (2015). Paraoxonase 1 activity and survival in Sepsis patients. Balkan Med J.

[24] Shukeri WFWM, Ralib AM, Abdulah NZ, Mat-Nor MB (2018). Sepsis mortality score for the prediction of mortality in septic patients. J Crit Care.

[25] Torrente C, Manzanilla EG, Bosch L, Villaverde C, Pastor J, de Gopegui RR (2019). The diagnostic and prognostic value of paraoxonase-1 and butyrylcholinesterase activities compared with acute-phase proteins in septic dogs and stratified by the acute patient physiologic and laboratory evaluation score. Vet Clin Pathol.

[26] Van Lenten BJ, Reddy ST, Navab M, Fogelman AM (2006). Understanding changes in high density lipoproteins during the acute phase response. Arterioscler Thromb Vasc Biol.

[27] Feingold KR, Memon RA, Moser AH, Grunfeld C (1998). Paraoxonase activity in the serum and hepatic mRNA levels decrease during the acute phase response. Atherosclerosis..

[28] Ruggerone B, Bonelli F, Nocera I, Paltrinieri S, Giordano A, Sgorbini M (2018). Validation of a paraoxon-based method for measurement of paraoxonase (PON-1) activity and establishment of RIs in horses. Vet Clin Pathol.

[29] Giordano A, Veronesi MC, Rossi G, Pezzia F, Probo M, Giori L (2013). Serum paraoxonase-1 activity in neonatal calves: age related variations and comparison between healthy and sick animals. Vet J.

[30] Iftimie S, Escribano A, Díez-Sans A, Albiciuc I, Hernández-Aguilera A, Fort-Gallifa I, et al. Influence of surgical procedures on serum Paraoxonase-1-related variables and markers of inflammation in hospitalized patients. J Investig Surg Off J Acad Surg Res. 2019. p. 1–9. 10.1080/08941939.2019.1597223. 10.1080/08941939.2019.1597223 30947571

[31] Oliveira-Filho JP, Badial PR, Cunha PHJ, Peiró JR, Araújo JPJ, Divers TJ (2012). Lipopolysaccharide infusion up-regulates hepcidin mRNA expression in equine liver. Innate Immun.

[32] Roy M-F, Kwong GPS, Lambert J, Massie S, Lockhart S (2017). Prognostic value and development of a scoring system in horses with systemic inflammatory response syndrome. J Vet Intern Med.

[33] James RW, Deakin SP (2004). The importance of high-density lipoproteins for paraoxonase-1 secretion, stability, and activity. Free Radic Biol Med.

[34] Bonelli F, Meucci V, Divers TJ, Wagner B, Intorre L, Sgorbini M (2017). Kinetics of plasma procalcitonin, soluble CD14, CCL2 and IL-10 after a sublethal infusion of lipopolysaccharide in horses. Vet Immunol Immunopathol.

[35] Sheats MK (2019). A comparative review of equine SIRS, Sepsis, and neutrophils. Front Vet Sci.

[36] Fogle J, Jacob M, Blikslager A, Edwards A, Wagner B, Dean K (2017). Comparison of lipopolysaccharides and soluble CD14 measurement between clinically endotoxaemic and nonendotoxaemic horses. Equine Vet J.

[37] Nieto J, MacDonald MH, Poulin Braim AE, Aleman MR (2009). Effect of lipopolysaccharide infusion on gene expression of inflammatory cytokines in normal horses in vivo. Equine Vet J.

[38] Poulin Braim AE, MacDonald MH, Bruss ML, Grattendick KJ, Giri SN, Margolin SB (2009). Effects of intravenous administration of pirfenidone on horses with experimentally induced endotoxemia. Am J Vet Res.

[39] Long A, Nolen-Walston R (2020). Equine inflammatory markers in the twenty-first century: a focus on serum amyloid a. Vet Clin North Am Equine Pract.

[40] Nunokawa Y, Fujinaga T, Taira T, Okumura M, Yamashita K, Tsunoda N (1993). Evaluation of serum amyloid a protein as an acute-phase reactive protein in horses. J Vet Med Sci.

[41] Jacobsen S, Thomsen MH, Nanni S (2006). Concentrations of serum amyloid a in serum and synovial fluid from healthy horses and horses with joint disease. Am J Vet Res.

[42] Taylor S (2015). A review of equine sepsis. Equine Vet Educ.

[43] Witkowska-Piłaszewicz OD, Żmigrodzka M, Winnicka A, Miśkiewicz A, Strzelec K, Cywińska A (2019). Serum amyloid a in equine health and disease. Equine Vet J.

[44] Van Lenten BJ, Hama SY, de Beer FC, Stafforini DM, McIntyre TM, Prescott SM (1995). Anti-inflammatory HDL becomes pro-inflammatory during the acute phase response. Loss of protective effect of HDL against LDL oxidation in aortic wall cell cocultures. J Clin Invest.

[45] Kotani K, Yamada T, Gugliucci A. Paired measurements of paraoxonase 1 and serum amyloid a as useful disease markers. Biomed Res Int. 2013. 10.1155/2013/481437. 10.1155/2013/481437 PMC3818810 24228251

[46] Han CY, Chiba T, Campbell JS, Fausto N, Chaisson M, Orasanu G (2006). Reciprocal and coordinate regulation of serum amyloid a versus apolipoprotein A-I and paraoxonase-1 by inflammation in murine hepatocytes. Arterioscler Thromb Vasc Biol.

[47] Tadros EM, Frank N (2012). Effects of continuous or intermittent lipopolysaccharide administration for 48 hours on the systemic inflammatory response in horses. Am J Vet Res.

[48] Iftimie S, García-Heredia A, Pujol I, Ballester F, Fort-Gallifa I, Simó JM (2016). A preliminary study of paraoxonase-1 in infected patients with an indwelling central venous catheter. Clin Biochem.

[49] Rossi G, Giordano A, Pezzia F, Kjelgaard-Hansen M, Paltrinieri S (2013). Serum paraoxonase 1 activity in dogs: preanalytical and analytical factors and correlation with C-reactive protein and alpha-2-globulin. Vet Clin Pathol.

[50] Borges AS, Divers TJ, Stokol T, Mohammed OH (2007). Serum iron and plasma fibrinogen concentrations as indicators of systemic inflammatory diseases in horses. J Vet Intern Med.

[51] Moore JN, Norton N, Barton MH, Hurley DJ, Reber AJ, Donovan DC (2007). Rapid infusion of a phospholipid emulsion attenuates the effects of endotoxaemia in horses. Equine Vet J.

