# Supplementary material for: Serial measurements of Paraoxonase-1 (PON-1) activity in horses with experimentally induced endotoxemia
Source: BMC Vet Res. 2020 Nov 4;16:422. doi: 10.1186/s12917-020-02629-4 (PMC7641807; doi:10.1186/s12917-020-02629-4)
Supplement: Supplementary file 1 — Additional file 1. Mean ± standard deviation regarding the clinical score and the main clinico-pathological changes recorded in the previous study [31] in the time points selected for inclusion in this study. [file 12917_2020_2629_MOESM1_ESM.docx]

Additional file 1: mean ± standard deviation regarding the clinical score and the main clinico-pathological changes recorded in the previous study (31) in the time points selected for inclusion in this study

| Time | WBC  x 10^3^/µL | PMN  x 10^3^/µL | PCV  (%) | Hb  (g/dL) | Iron  (μg/dL) | Fibrinogen  (mg/dL) | Tot protein  (g/dL) | CLINICAL  SCORE |
| --- | --- | --- | --- | --- | --- | --- | --- | --- |
| 0 | 8.3 ± 1.2 | 4.4 ± 0.8 | 36,5 ± 2,7 | 10.8 ± 2.1 | 105.2 ± 15.0 | 300.0 ± 63.2 | 7.2 ± 0.4 | 0.00 ± 0.00 |
| 2 | Nd | Nd | 36,3 ± 2,9 | Nd | 103.4 ± 17.8 | 250.0 ± 54.8 | 7.1 ± 0.4 | 1.67 ± 0.26 |
| 6 | 9.8 ± 3.0 | 8.0 ± 0.3 | 37,2 ± 3,0 | 12.4 ± 1.3 | 70.5 ± 10.4 | 300.0 ± 89.4 | 7.4 ± 0.5 | 0.67 ± 0.27 |
| 12 | nd | Nd | 36,8 ± 3,3 | Nd | 36.4 ± 4.9 | 283.3 ± 160.2 | 7.4 ± 0.3 | 0.00 ± 0.00 |
| 24 | 13.3 ± 2.7 | 9.1 ± 2.5 | 35,8 ± 4,1 | 11.5 ± 1.5 | 66.7 ± 31.7 | 350.0 ± 54.8 | 7.2 ± 0.4 | 0.00 ± 0.00 |
| 36 | Nd | Nd | 35,3 ± 3,7 | Nd | 142.6 ± 37.8 | 383.3 ± 147.2 | 7.2 ± 0.5 | 0.00 ± 0.00 |
| 48 | 11.9 ± 1.4 | 7.4 ± 1.5 | 35,3 ± 2,3 | 11.5 ± 1.2 | 146.5 ± 22.8 | 350.0 ± 104.9 | 7.1 ± 0.4 | 0.00 ± 0.00 |
| 72 | 11.2 ± 2.1 | 6.6 ± 1.2 | 35,3 ± 1,8 | 11.3 ± 1.0 | 115.9 ± 30.5 | 383.3 ± 132.9 | 7.2 ± 0.6 | 0.00 ± 0.00 |
| 168 | 9.5 ± 1.1 | 5.4 ± 0.9 | 35,7 ± 2,9 | 11.6 ± 1.0 | 113.1 ± 28.5 | 366.7 ± 103.3 | 7.5 ± 0.7 | 0.00 ± 0.00 |
| 240 | 9.2 ± 1.1 | 4.7 ± 1.1 | 35,5 ± 2,1 | 11.6 ± 1.2 | 102.6 ± 20.5 | 316.7 ± 98.3 | 7.7 ± 0.5 | 0.00 ± 0.00 |
